# The *Staurotypus* Turtles and Aves Share the Same Origin of Sex Chromosomes but Evolved Different Types of Heterogametic Sex Determination

**DOI:** 10.1371/journal.pone.0105315

**Published:** 2014-08-14

**Authors:** Taiki Kawagoshi, Yoshinobu Uno, Chizuko Nishida, Yoichi Matsuda

**Affiliations:** 1 Laboratory of Animal Genetics, Department of Applied Molecular Biosciences, Graduate School of Bioagricultural Sciences, Nagoya University, Nagoya, Japan; 2 Department of Natural History Sciences, Faculty of Science, Hokkaido University, Sapporo, Japan; 3 Avian Bioscience Research Center, Graduate School of Bioagricultural Sciences, Nagoya University, Nagoya, Japan; University of Florence, Italy

## Abstract

Reptiles have a wide diversity of sex-determining mechanisms and types of sex chromosomes. Turtles exhibit temperature-dependent sex determination and genotypic sex determination, with male heterogametic (XX/XY) and female heterogametic (ZZ/ZW) sex chromosomes. Identification of sex chromosomes in many turtle species and their comparative genomic analysis are of great significance to understand the evolutionary processes of sex determination and sex chromosome differentiation in Testudines. The Mexican giant musk turtle (*Staurotypus triporcatus*, Kinosternidae, Testudines) and the giant musk turtle (*Staurotypus salvinii*) have heteromorphic XY sex chromosomes with a low degree of morphological differentiation; however, their origin and linkage group are still unknown. Cross-species chromosome painting with chromosome-specific DNA from Chinese soft-shelled turtle (*Pelodiscus sinensis*) revealed that the X and Y chromosomes of *S*. *triporcatus* have homology with *P. sinensis* chromosome 6, which corresponds to the chicken Z chromosome. We cloned cDNA fragments of *S*. *triporcatus* homologs of 16 chicken Z-linked genes and mapped them to *S*. *triporcatus* and *S*. *salvinii* chromosomes using fluorescence in situ hybridization. Sixteen genes were localized to the X and Y long arms in the same order in both species. The orders were also almost the same as those of the ostrich (*Struthio camelus*) Z chromosome, which retains the primitive state of the avian ancestral Z chromosome. These results strongly suggest that the X and Y chromosomes of *Staurotypus* turtles are at a very early stage of sex chromosome differentiation, and that these chromosomes and the avian ZW chromosomes share the same origin. Nonetheless, the turtles and birds acquired different systems of heterogametic sex determination during their evolution.

## Introduction

The constitutions of sex chromosome and sex-determination systems of reptiles are extraordinarily diverse. Reptiles exhibit both genotypic sex determination (GSD)– in which the sex of offspring is determined by a sex-determining gene on the sex chromosome – and temperature-dependent sex determination (TSD)– in which the sex ratio depends on the incubation temperature of embryos. GSD systems are found in all snakes, many lizards, and a small number of turtles [1]. Almost all snakes exhibit female heterogamety (ZZ/ZW), whereas lizards and turtles with GSD exhibit both male heterogamety (XX/XY) and female heterogamety [2]. In lizards, sex chromosomes have been identified for 181 species, with 115 species shown to exhibit male heterogamety and 66 shown to exhibit female heterogamety [3]. Given that the distribution of XY and ZW species shows no clear phylogenetic segregation [3]–[6], it seems likely that the sex chromosomes differentiated independently in each lineage. On the other hand, whereas 18 turtle species from the order Testudines exhibit GSD [7], differentiated sex chromosomes have been identified for only nine such species (six XX/XY species and three ZZ/ZW species) [2], [7]–[12]. Among them, the ZW sex chromosomes of Chinese soft-shelled turtle (*Pelodiscus sinensis*, Trionychidae) have conserved linkage homology with chicken chromosome 15 [11], whereas the XY sex chromosomes of the black marsh turtle (*Siebenrockiella crassicollis*, Geoemydidae) share linkage homology with chicken chromosome 5 [12]. These results suggest that the sex chromosomes of birds and turtles differentiated from different autosomal pairs of the common ancestor of Archosauromorpha, which diverged 250–270 million years ago (MYA) [13]–[15]. The group Archosauromorpha contains Archosauria (diapsid amniotes whose living representatives consist of birds and crocodilians) and all other saurians that are closer to Archosauria than they are to Lepidosauria (including tuataras, lizards, snakes and amphisbaenia). However, the origins of the sex chromosomes of the other seven GSD turtle species with differentiated sex chromosomes are still unknown. Identification of the linkage groups of the sex chromosomes and their homologies in other reptilian and avian species will improve our understanding of the evolutionary mechanisms that drive the genetic determination of sex and the differentiation of sex chromosomes in extant vertebrates.

The Mexican giant musk turtle (*Staurotypus triporcatus*, Kinosternidae) and the giant musk turtle (*Staurotypus salvinii*) inhabit the region from eastern and southern North America to Argentina and have heteromorphic X and Y sex chromosomes [16], [17]. The X and Y chromosomes were only slightly different in terms of the sizes of the short arms and secondary constrictions in the two species, as determined by conventional Giemsa staining. Neither the structural differences between the X and Y chromosomes at the molecular level nor their linkage groups have been determined. The present study involved comparative mapping of functional genes for the X and Y chromosomes of *S. triporcatus* and *S*. *salvinii* in order to elucidate the origin and evolution of the sex chromosomes of *Staurotypus* turtles. The homology of the X chromosomes of *Staurotypus* turtles with the chicken Z chromosome was found by cross-species hybridization with chromosome paints of Chinese soft-shelled turtle (*Pelodiscus sinensis*); therefore, we isolated *S. triporcatus* homologs of 16 chicken Z-linked genes and mapped them to chromosomes of *S. triporcatus* and *S*. *salvinii*. Comparison of the cytogenetic maps of the X chromosomes of these two turtle species with that of the Z chromosome of the ostrich (*Struthio camelus*), which is one of the most primitive extant avian species and retains the ancestral type of avian Z chromosomes, sheds light on the differentiation of the X and Y chromosomes of *Staurotypus* turtles and the evolution of sex chromosomes in Testudines.

## Materials and Methods

### Cell culture and chromosome preparation

For each of *S. triporcatus* and *S. salvinii*, a male that had been bred in captivity was purchased and used for this study. After intra-peritoneal injection of a fatal dose of pentobarbital, the heart, lung, and mesentery were removed and used for cell culture at 26°C in a humidified atmosphere of 5% CO_2_ in air. Animal care and all experimental procedures were approved by the Animal Experiment Committee, Graduate School of Bioagricultural Sciences, Nagoya University (approval no. 2010052401), and the experiments were conducted according to the Regulations on Animal Experiments in Nagoya University. Cell culturing and chromosome preparation were performed as described previously [12]. Fibroblasts of the ostrich used in our previous study [18] were recovered from liquid nitrogen and subsequently cultured for chromosome preparation. For gene mapping by fluorescence in situ hybridization (FISH), replication banding was performed to identify each chromosome precisely, as described previously [12], [19]. The fibroblast cell cultures were treated with BrdU (12 µg/ml) (Sigma-Aldrich) at the late replication stage for 12 h, including 45 min of colcemid treatment, and chromosome preparations were made using an air-drying method. The cultured cells of the ostrich were harvested after 6 h of treatment with BrdU (25 µg/ml) under conditions of 39°C with 5% CO_2_ in air. After staining the slides with Hoechst 33258 (1 µg/ml) for 10 min, replication bands were obtained by heating them at 65°C for 3 min and exposing them to UV light at 65°C for an additional 6.5 min. The slides were kept at –80°C until use.

### C-banding

To examine the chromosomal distribution of constitutive heterochromatin in *S. triporcatus* and *S. salvinii*, C-banding was performed by the standard barium hydroxide/saline/Giemsa method [20] with slight modification; chromosome slides were treated with 0.2N HCl at room temperature for 5 min and then 5% Ba (OH)_2_ at 50°C for 2 min.

### Chromosome painting

Cross-species chromosome painting with chromosome-specific DNA probes of *P. sinensis* was performed for *S. triporcatus.* The *P. sinensis* chromosome paints were prepared and provided by Fengtang Yang and Patricia O’Brien, both from the Department of Veterinary Medicine, Cambridge University, UK. Chromosome painting was performed as described previously [12], [21]. One microgram of DNA probe was labeled with biotin-16-dUTP (Roche Diagnostics) using a nick translation kit (Roche Diagnostics). After pre-hybridization for 15 min at 37°C, hybridization was carried out at 37°C for five days. After hybridization, the slide was washed, incubated with fluorescein-conjugated avidin (Roche Diagnostics), and stained with 0.75 µg/ml propidium iodide (PI).

### Molecular cloning of *S. triporcatus* and ostrich homologs of chicken genes

Testis and brain of *S*. *triporcatus* and testis of the ostrich were homogenized and lysed with TRIzol Reagent (Life Technologies), and total RNA was extracted following the manufacturer’s instructions. Testis tissues of the ostrich used in our previous study [18] were recovered from liquid nitrogen. Molecular cloning of *S*. *triporcatus* and ostrich homologs of the chicken Z-linked genes was performed by reverse transcription polymerase chain reaction (RT-PCR) using the PCR primers shown in Table S1. The nucleotide sequences of cDNA fragments were determined and compared as described previously [22].

### FISH mapping

FISH was performed for chromosomal localization of the 18S–28S ribosomal RNA (rRNA) genes and cDNA fragments of functional genes as described by Kawagoshi et al. [11] and Matsuda and Chapman [19]. After FISH of the rRNA genes, Ag-NOR staining was performed to visualize nucleolar organizing regions (NORs) on the same metaphase spreads following Howell and Black [23]. For chromosome mapping of functional genes, 250 ng of cDNA fragments were labeled with biotin-16-dUTP (Roche Diagnostics) by nick translation. After hybridization, the probe DNA was hybridized with goat anti-biotin antibody (Vector Laboratories), stained with Alexa Fluor 488 rabbit anti-goat IgG (H+L) conjugate (Life Technologies-Molecular Probes), and then counter-stained with 0.75 µg/ml PI.

## Results

### Karyotypes of *S. triporcatus* and *S. salvinii*

**Figure 1:**
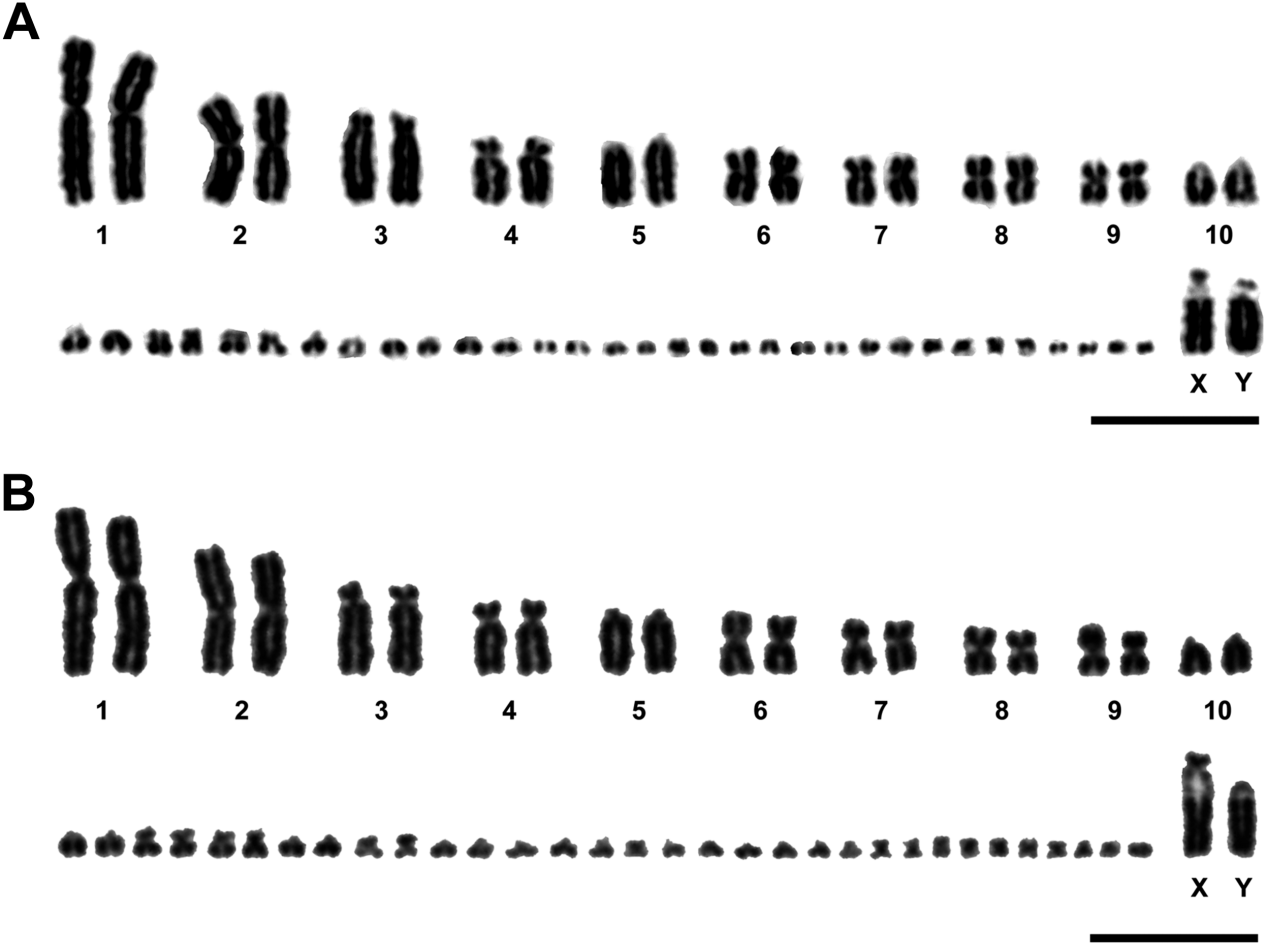
Giemsa-stained karyotypes of male *S. triporcatus* and *S. salvinii*. (A) *S. triporcatus.* (B) *S. salvinii.* The X and Y chromosomes have large and small secondary constrictions, respectively. Scale bars = 10 µm.

Twenty Giemsa-stained metaphase spreads of *S*. *triporcatus* and 18 metaphase spreads of *S. salvinii* were examined for karyotyping. The chromosome numbers were 2n = 54 in all metaphase spreads of both species, as reported previously [16]. Karyotypes of both species consisted of four pairs of large chromosomes including sex chromosomes (chromosomes 1–3 and X and Y chromosomes), seven pairs of medium-sized and/or small chromosomes (chromosomes 4–10), and 16 pairs of indistinguishable microchromosomes (Figure 1). The sex chromosomes were morphologically differentiated: whereas the X chromosomes were acrocentric in *S. triporcatus* and subtelocentric in *S. salvinii*, with a secondary constriction on the long arm near the centromere, the Y chromosomes were both acrocentric; and the size of the secondary constriction was larger in the X chromosomes than in the Y chromosomes.

**Figure 2:**
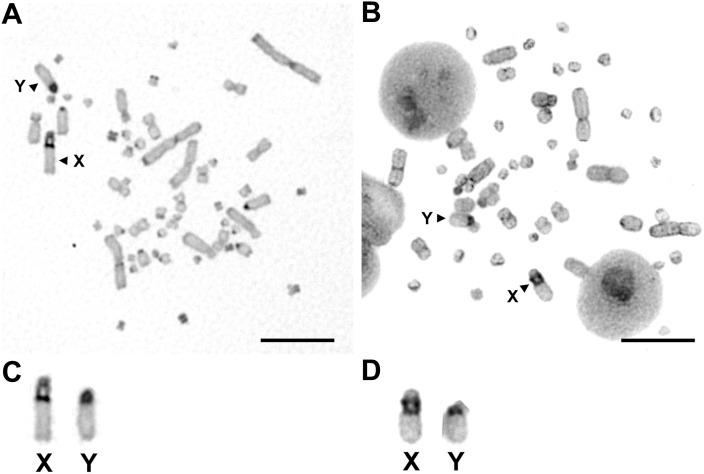
C-banded metaphase spreads of male *S. triporcatus* and *S*. *salvinii*. (A) *S. triporcatus.* (B) *S. salvinii.* (C, D) Enlarged photographs of the X and Y chromosomes of *S. triporcatus* (C) and *S*. *salvinii* (D). Scale bars = 10 µm.

C-positive heterochromatin blocks were observed in the centromeric regions of almost all autosomes and the telomeric regions of several pairs of autosomes in both species (Figures 2A, B). Chromosomal regions surrounding the secondary constrictions on the X and Y chromosomes were heterochromatized and showed C-positive bands in both species (Figures 2C, D).

### Chromosomal locations of the 18S-28S rRNA genes and NORs in *S. triporcatus* and *S. salvinii*

**Figure 3:**
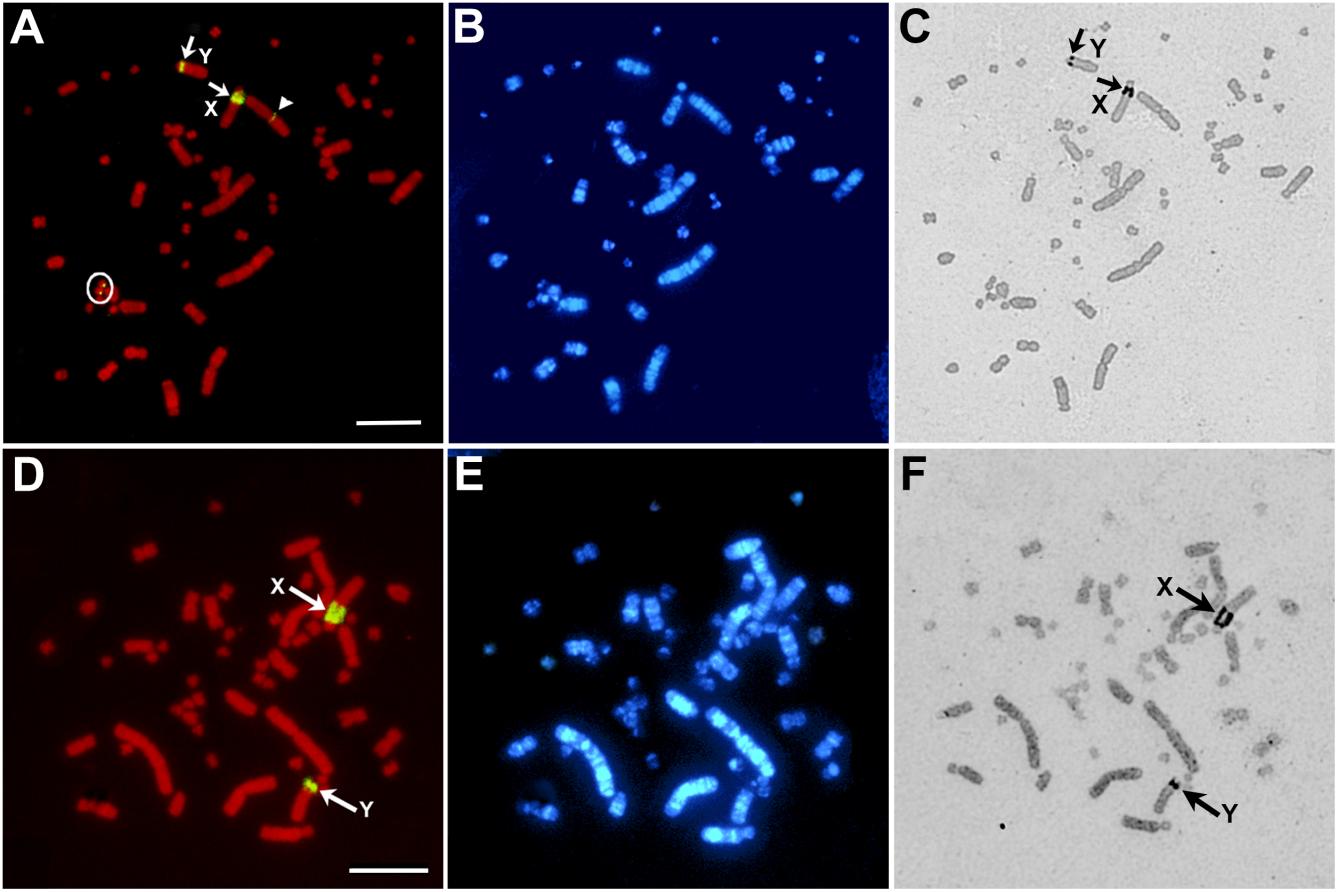
Chromosomal distribution of the 18S-28S rRNA genes and NORs on metaphase spreads of male *S*. *triporcatu*s and *S*. *salvinii*. (A–C) *S*. *triporcatu*s. (D–F) *S*. *salvinii*. FISH signals of the 18S–28S rRNA genes were localized to the secondary constrictions of the X and Y chromosomes (indicated by arrows), one of the copies of chromosome 2 (an arrowhead), and a pair of microchromosomes (a circle) in *S*. *triporcatus* (A), and the secondary constrictions of the X and Y chromosomes in *S*. *salvinii* (D). Ag-stained NORs were also distributed in the secondary constrictions of the X and Y chromosomes in *S*. *triporcatus* (C) and *S*. *salvinii* (F). However, no NORs were detected on chromosome 2 and a pair of microchromosomes in *S*. *triporcatus*, where the FISH signals of the rRNA genes were detected. (B, E) Hoechst-stained patterns of the same PI-stained metaphase spreads (A) and (D), respectively. Scale bars = 10 µm.

FISH signals of the 18S–28S rRNA genes were detected in the secondary constrictions of the X and Y chromosomes, one of the copies of chromosome 2, and a pair of microchromosomes in *S. triporcatus* (Figure 3A). In *S. salvinii*, signals were detected only in the secondary constrictions of the X and Y chromosomes (Figure 3D). There was a remarkable difference in the size of hybridization signals between the X and Y chromosomes in both species, which corresponded to the difference in the size of secondary constrictions. NORs were detected in the secondary constrictions of the X and Y chromosomes in both species using Ag-NOR staining, whereas no NORs were found for chromosome 2 and a pair of microchromosomes in *S. triporcatus* (Figures 3C, F), in which small FISH signals of rRNA genes were observed (Figure 3A).

### Chromosome homology of the *S. triporcatus* X chromosome with the chicken Z chromosome

**Figure 4:**
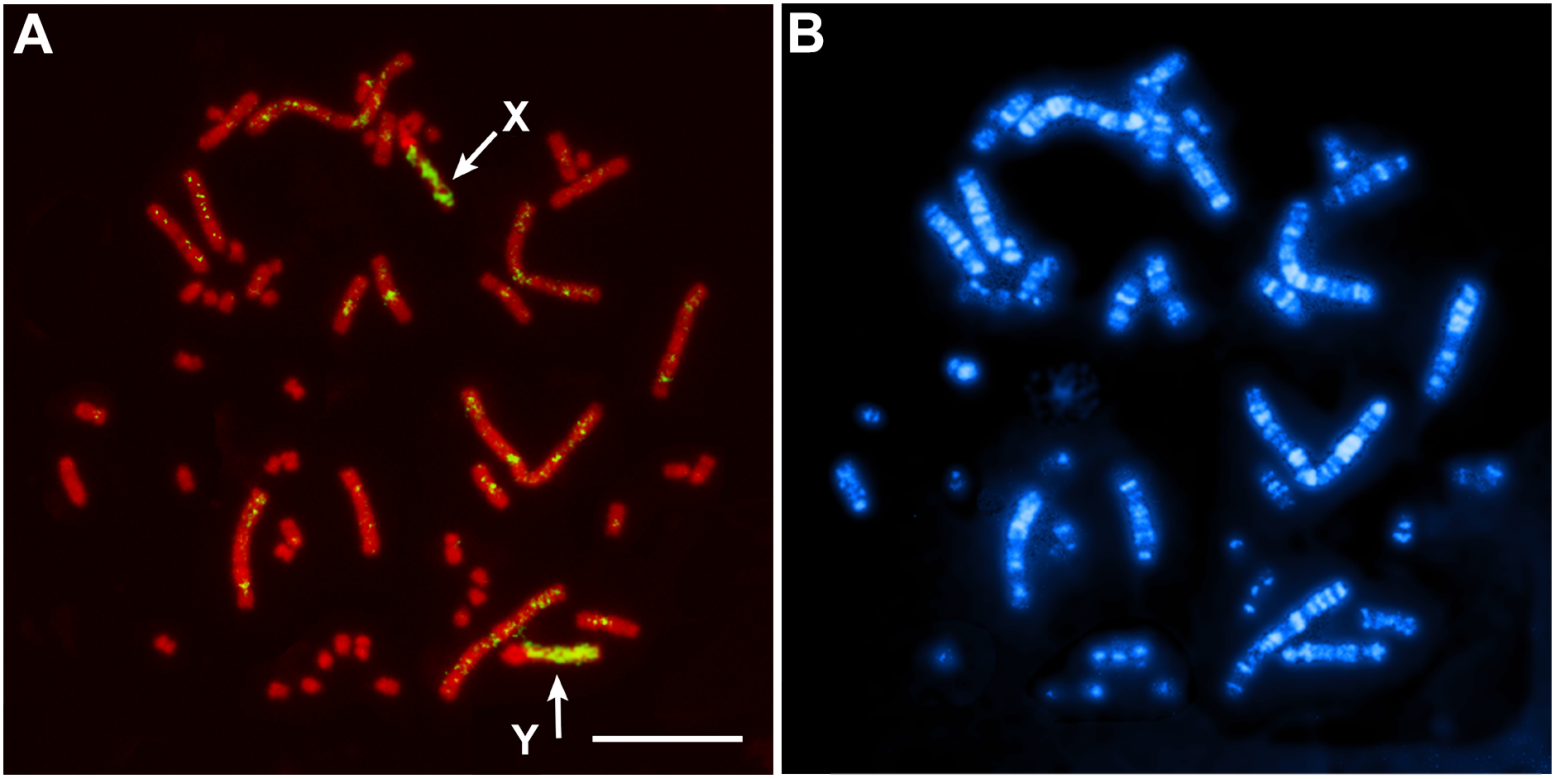
Chromosome painting with chromosome 6-specific DNA probe of *P. sinensis* to metaphase spread of male *S. triporcatus*. (A) The probe painted the X and Y chromosomes on PI-stained metaphase spread of *S. triporcatus* (indicated by arrows). (B) Hoechst-stained pattern of the same metaphase spread as in (A). Scale bar = 10 µm.

Hybridization of the chromosome 6 paint of *P. sinensis* to the X and Y chromosomes of *S. triporcatus* (Figure 4) indicated that the *S. triporcatus* X and Y sex chromosomes are a counterpart of *P. sinensis* chromosome 6, which is homologous to the chicken Z chromosome [24], [25].

### Chromosomal locations of *S. triporcatus* homologs of chicken Z-linked genes

**Figure 5:**
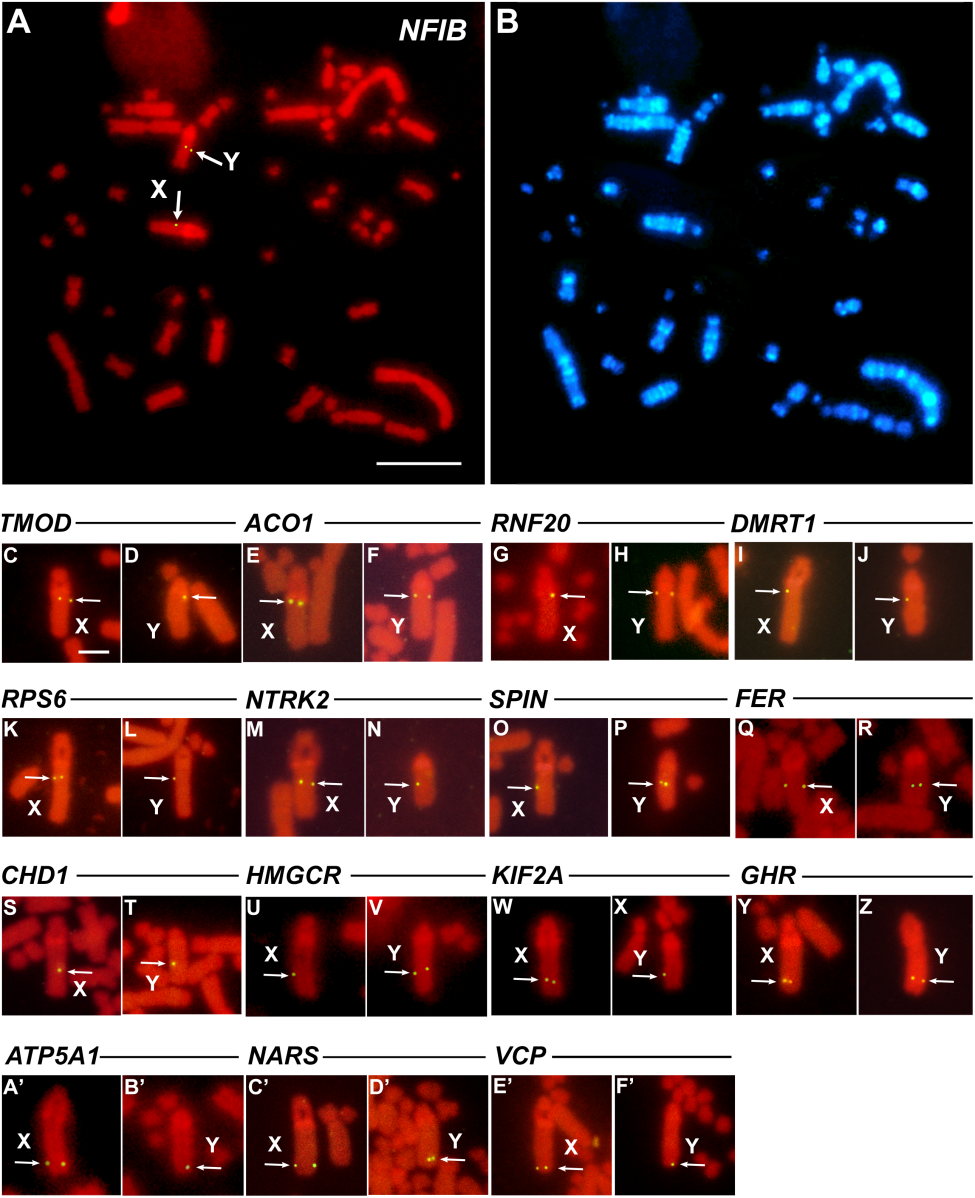
Chromosomal locations of *S. triporcatus* homologs of 16 chicken Z-linked genes in male *S. triporcatus*. (A, B) FISH pattern of *NFIB* on PI-stained metaphase spread (A) and Hoechst-stained pattern of the same metaphase spread (B). (C–Z, A’–F’) FISH signals of *TMOD* (C, D), *ACO1* (E, F), *RNF20* (G, H), *DMRT1* (I, J), *RPS6* (K, L), *NTRK2* (M, N), *SPIN* (O, P), *FER* (Q, R), *CHD1* (S, T), *HMGCR* (U, V), *KIF2A* (W, X), *GHR* (Y, Z), *ATP5A1* (A’, B’), *NARS* (C’, D’), and *VCP* (E’, F’) on PI-stained X and Y chromosomes. Arrows indicate the hybridization signals of the genes. Scale bars represent 10 µm (A, B) and 2.5 µm (C–Z, A’–F’).

**Figure 6:**
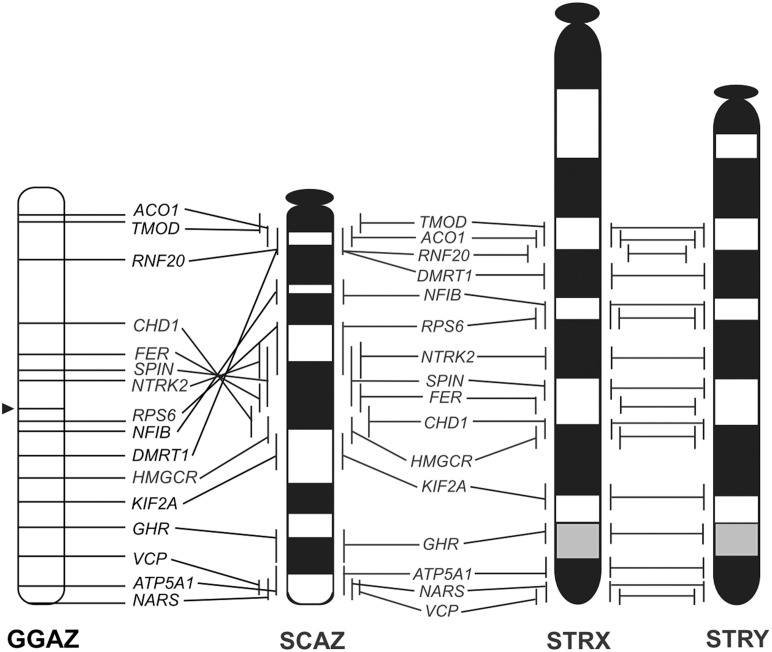
Comparative cytogenetic maps of 16 functional genes on the chicken Z chromosome (GGAZ), the ostrich Z chromosome (SCAZ), and the X and Y chromosomes of *S. triporcatus* (STRX and STRY, respectively). The gene order of 16 genes on the chicken Z chromosome was taken from the Ensembl Chicken Genome Browser (http://www.ensembl.org/Gallus_gallus). The chicken Z chromosome is inverted to facilitate comparison of the gene order. Arrowhead represents the location of the centromere.

On the basis of the result that *S. triporcatus* X and Y sex chromosomes are homologous to the chicken Z chromosome, we cloned *S. triporcatus* homologs of 16 chicken Z-linked genes: *ACO1*, *ATP5A1*, *CHD1*, *DMRT1*, *FER*, *GHR, HMGCR*, *KIF2A*, *NARS*, *NFIB*, *NTRK2*, *RNF20*, *RPS6*, *SPIN*, *TMOD*, and *VCP*. Nucleotide sequence identities in the equivalent regions of cDNA fragments of these 16 genes between *S. triporcatus* and chicken ranged from 77.7% to 94.4% (Table 1). Hoechst-stained bands obtained by the replication banding method enabled precise determination of the subchromosomal locations of the genes (Figure 5). For FISH mapping, 25–30 metaphase spreads were observed for each gene. The hybridization efficiency ranged from 20% to 36% on the X chromosome, and from 23% to 38% on the Y chromosome. Sixteen homologs of chicken Z-linked genes were all localized to the long arm of *S. triporcatus* X and Y chromosomes in the same order (Figure 6).

**Table 1:** The cDNA fragments of *S. triporcatus* (STR) homologs of chicken Z-linked genes and nucleotide sequence identities between *S. triporcatus* and chicken (*Gallus gallus*, GGA) cDNA fragments.

Gene^a^	Length of cDNAfragment (bp)	Identity (%) betweenSTR and GGA^b^	Accession numberof *S. triporcatus* homolog
*ACO1*	1135	83.1 (943/1135)	AB747261
*ATP5A1*	1102	86.8 (956/1102)	AB747262, AB747263
*CHD1*	893	88.8 (793/893)	AB747264
*DMRT1*	684	81.2 (553/681)	AB747265
*FER*	760	91.4 (695/760)	AB747266
*GHR*	898	77.7 (698/898)	AB747267
*HMGCR*	1077	84.4 (909/1077)	AB747268
*KIF2A*	664	93.2 (619/664)	AB747269
*NARS*	1083	85.0 (921/1083)	AB747271
*NFIB*	820	94.4 (774/820)	AB747272
*NTRK2*	554	89.7 (497/554)	AB747273
*RNF20*	1159	84.9 (984/1159)	AB747274
*RPS6*	658	86.4 (569/658)	AB747275
*SPIN*	628	93.3 (586/628)	AB747276
*TMOD*	1007	82.2 (828/1007)	AB747277
*VCP*	995	90.1 (897/995)	AB747278

a *ACO1*, aconitase 1, soluble; *ATP5A1*, ATP synthase, H^+^ transporting, mitochondrial F1 complex, alpha subunit, isoform 1, cardiac muscle; *CHD1*, chromodomain helicase DNA binding protein 1; *DMRT1*, *doublesex* and *mab-3* related transcription factor 1; *FER*, (fps/fes related) tyrosine kinase; *GHR*, growth hormone receptor; *HMGCR*, 3-hydroxy-3-methylglutaryl-CoA reductase; *KIF2A*, kinesin heavy chain member 2A; *NARS*, asparaginyl-tRNA synthetase; *NFIB*, nuclear factor I/B; *NTRK2*, neurotrophic tyrosine kinase receptor, type 2; *RNF20*, ring finger protein 20, E3 ubiquitin protein ligase; *RPS6*, ribosomal protein S6; *SPIN*, spindlin; *TMOD*, tropomodulin 1; *VCP*, valosin containing protein.

b The number in parenthesis indicates the number of identical bases/the number of bases in the overlapped region between cDNA fragments of two species.

### Comparison between the *S. triporcatus* X chromosome and the ostrich Z chromosome

We cloned ostrich homologs of eight chicken Z-linked genes, *ACO1*, *FER*, *HMGCR*, *KIF2A*, *NARS*, *NFIB*, *RNF20*, and *VCP*, by RT-PCR using the PCR primers shown in Table S1 and mapped them to ostrich chromosomes by FISH (Figure S1). Although *ACO1* (*IREBP1*) was previously mapped to the ostrich Z chromosome [26], [27], we cloned a cDNA fragment of this gene and mapped it to determine its precise location on the ostrich Z chromosome. We also mapped *DMRT1* to ostrich chromosomes using the cDNA fragments isolated in our previous study [18]. We then constructed a cytogenetic map of the ostrich Z and W chromosomes with 16 functional genes by adding seven ostrich Z-linked genes (*ATP5A1*, *CHD1*, *GHR*, *NTRK2*, *RPS6*, *SPIN*, and *TMOD*), which were cloned and mapped in our previous studies (Figure S2) [18], [27]. Nucleotide sequence identities in the equivalent regions of cDNA fragments of 16 genes ranged from 79.6% to 94.4% between *S. triporcatus* and the ostrich (Table 2). In general, the identities of nucleotide sequences were higher in 14 genes than in those between *S*. *triporcatus* and chicken; exceptions were for *NFIB* and *VCP*, for which the nucleotide sequence identities did not differ (Tables 1 and 2). Eleven genes (*RPS6*, *NTRK2*, *SPIN*, *FER*, *CHD1*, *HMGCR*, *KIF2A*, *GHR*, *ATP5A1*, *NARS*, and *VCP*) were localized to the ostrich Z and W chromosomes in the same order, whereas five genes (*TMOD*, *ACO1*, *RNF20*, *DMRT1*, and *NFIB*) were not mapped to the W chromosome (Figure S2). This indicated that the proximal region of the ostrich Z chromosome that contained these five genes had been deleted in the W chromosome. The order of 16 genes on the ostrich Z chromosome was almost the same as those on the X and Y chromosomes of *S. triporcatus* (Figure 6), although the precise order among several genes located close together was not determined.

**Table 2:** The cDNA fragments of ostrich (*S. camelus*, SCA) homologs of chicken Z-linked genes and nucleotide sequence identities among *S. triporcatus* (STR), ostich and chicken (*Gallus gallus*, GGA) cDNA fragments.

Gene^a^	Length of cDNAfragment (bp)	Identity (%) betweenSTR and SCA^b^	Identity (%) betweenSCA and GGA^b^	Accession numberof ostrich homolog
*ACO1*	1133	83.6 (948/1133)	91.7 (1039/1133)	AB755561
*ATP5A1*	990	88.1 (873/990)	92.5 (916/990)	AB254864^c^, AB254866^c^
*CHD1*	874	89.4 (780/872)	92.1 (805/874)	AB254867^c^
*DMRT1*	1262	87.1 (420/482)	88.3 (575/651)	AB536738^d^
*FER*	761	92.5 (703/760)	94.3 (718/761)	AB747279
*GHR*	832	79.6 (653/820)	86.8 (712/820)	AB254871^c^
*HMGCR*	1074	85.7 (920/1074)	91.6 (984/1074)	AB747280
*KIF2A*	666	93.8 (623/664)	95.8 (637/665)	AB747281
*NARS*	1085	86.0 (931/1083)	91.7 (994/1084)	AB747283
*NFIB*	820	94.4 (774/820)	95.2 (781/820)	AB747284
*NTRK2*	500	90.8 (454/500)	94.8 (474/500)	AB254873^c^
*RNF20*	1171	86.4 (999/1156)	91.8 (1076/1171)	AB747285
*RPS6*	612	87.3 (534/612)	93.8 (574/612)	AB254876^c^
*SPIN*	580	94.3 (547/580)	97.8 (567/580)	AB254878^c^
*TMOD*	901	83.9 (756/901)	90.1 (812/901)	AB254879^c^
*VCP*	995	90.0 (896/995)	93.4 (929/995)	AB747356

a *ACO1*, aconitase 1, soluble; *ATP5A1*, ATP synthase, H^+^ transporting, mitochondrial F1 complex, alpha subunit, isoform 1, cardiac muscle; *CHD1*, chromodomain helicase DNA binding protein 1; *DMRT1*, *doublesex* and *mab-3* related transcription factor 1; *FER*, (fps/fes related) tyrosine kinase; *GHR*, growth hormone receptor; *HMGCR*, 3-hydroxy-3-methylglutaryl-CoA reductase; *KIF2A*, kinesin heavy chain member 2A; *NARS*, asparaginyl-tRNA synthetase; *NFIB*, nuclear factor I/B; *NTRK2*, neurotrophic tyrosine kinase receptor, type 2; *RNF20*, ring finger protein 20, E3 ubiquitin protein ligase; *RPS6*, ribosomal protein S6; *SPIN*, spindlin; *TMOD*, tropomodulin 1; *VCP*, valosin containing protein.

b The number in parenthesis indicates the number of identical bases/the number of bases in the overlapped region between cDNA fragments of two species.

c The nucleotide sequences were obtained from Tsuda et al. [27].

d The nucleotide sequence was obtained from Ishijima et al. [18].

### Comparison of the XY chromosomes between *S. triporcatus* and *S. salvinii*

Sixteen genes were also all localized to the X and Y chromosomes of *S. salvinii*, and their locations and orders completely matched those of *S. triporcatus* (Figures S3 and S4). The hybridization efficiency ranged from 23% to 38% for 25–30 metaphase spreads.

## Discussion

The origin and evolutionary process of the X and Y sex chromosomes of *S. triporcatus* and *S. salvinii* were investigated using cross-species chromosome painting and chromosome mapping of cDNA clones of sex-linked genes isolated from *S. triporcatus*. Cross-species chromosome painting revealed that the X and Y chromosomes of *S. triporcatus* are homologous to *P. sinensis* chromosome 6, which corresponds to the chicken Z chromosome [24], [25]. The homology with the chicken Z chromosome has been also reported for the red-eared slider (*Trachemys scripta elegans*) chromosome 6 and Nile crocodile (*Crocodylus niloticus*) chromosome 6 [28]; however, the homology of these chromosomes with *P. sinensis* chromosome 6 is still not known.

*S. triporcatus* homologs of 16 chicken Z-linked genes were all shown to be localized to the long arm of the X and Y chromosomes of *S. triporcatus* and *S. salvinii* in the same order. These results suggest that the XY sex chromosomes of *Staurotypus* turtles share the same origin as avian ZW sex chromosomes; however; *Staurotypus* turtles and birds acquired different types of heterogametic sex-determination system during their evolution, and the X and Y chromosomes of *S. triporcatus* and *S. salvinii* are at a very early stage of differentiation. The only structural difference between the X and Y chromosomes in *S. triporcatus* was in the vicinity of the secondary constriction near the centromere, where meiotic recombination would have been suppressed. In *S. salvinii*, in addition to the difference in the size of the secondary constriction, the X and Y chromosomes were morphologically different: the X was subtelocentric, whereas the Y was acrocentric. The cessation of meiotic recombination very likely accounts for the difference in the copy number of the 18S–28S rRNA genes: this might have resulted from either a decrease in the copy number on the Y chromosome and/or amplification on the X chromosome. Alternatively, Sites et al. [17] suggested that the *S. salvinii* X chromosome was evolutionarily derived from the translocation of the NOR followed by the addition of a heterochromatic short arm onto the X, which occurred in one of the homomorphic proto-sex chromosomes, and the Y has remained unchanged. However, the initial step of sex chromosome differentiation in *Staurotypus* turtles remains unknown because the morphology of the homomorphic proto-sex chromosomes has not yet been identified.

The order of 16 genes on the *S. triporcatus* X chromosome was nearly identical to that of the ostrich Z chromosome, which bears the primitive gene order of avian sex chromosomes [27], [29] (Figure 6). This result suggests that the X chromosomes of *S*. *triporcatus* and *S*. *salvinii* and the ostrich Z chromosome are derived from the same autosomal pair of the common ancestor, and that the primitive gene order has been retained in both lineages independently since the time when Archosauromorpha diverged from the common ancestor of sauropsids 250–270 MYA [13]–[15]. In the chicken Z chromosome, the order was inverted in a region across the centromere, where seven genes (*DMRT1*, *NFIB*, *RPS6*, *NTRK2*, *SPIN*, *FER*, and *CHD1*) are contained, compared with those in the ostrich Z chromosome and the X chromosomes of two *Staurotypus* species. Moreover, the order of *DMRT1*–*NFIB*–*RPS6*–*NTRK2*–*SPIN*–*FER*–*CHD1* is probably the same as those of the ostrich Z and *Staurotypus* X chromosomes, although the location of the centromere differed (Figure 6). This result leads us to predict that a large paracentric inversion occurred at the breakpoints between *RNF20* and *DMRT1* and between *CHD1* and *HMGCR* in the ancestral acrocentric Z chromosome, and that subsequent repositioning of the centromere led to the metacentric chicken Z chromosome. Our previous studies revealed that whereas the ZW sex chromosomes of *P. sinensis* have homology with chicken chromosome 15, the XY chromosomes of *S. crassicollis* are homologous to chicken chromosome 5 [11], [12]. These results indicate that the sex chromosomes of these three turtle species differentiated independently from different autosomal pairs of the common ancestor in each lineage. This suggests great diversity of sex chromosomal origins and a considerable level of plasticity of sex determination in Testudines. Such diversity of sex chromosomal origins within the same order has also been found in squamate reptiles [22], [30]–[33]. The homology of the micro-X sex chromosome of the green anole lizard (*Anolis carolinensis*) to chicken chromosome 15 [31] indicates that *A*. *carolinensis* and *P. sinensis* happen to share the same origin of sex chromosomes. However, it remains unclear whether the gene order of the sex chromosomes has been conserved.

The family Kinosternidae is composed of two subfamilies, Staurotypinae and Kinosterninae [34]; however, molecular phylogenetic analysis has indicated that these two clades show monophyly within the family [35]. Staurotypinae comprises only three species: the narrow-bridged musk turtle (*Claudius angustatus*), *S*. *triporcatus*, and *S*. *salvinii*. These three species have similar karyotypes with 2n = 54. *C*. *angustatus* also exhibits GSD; however, this species has no heteromorphic sex chromosomes [16], [36]. The karyotypes of Kinosterninae species differ from those of Staurotypinae in terms of the diploid chromosome number (2n = 56), and no GSD species have been reported in this subfamily [2], [16], [37]. These observations collectively suggest that TSD was probably the primitive state in Kinosternidae and that GSD arose in the lineage of Staurotypinae; it thus seems likely that *Staurotypus* and *Claudius* share the ancestral XY sex chromosome system for this group but that *Claudius* remains at a more primitive stage of differentiation or that *Claudius* sex chromosomes are more recently derived than those in *Staurotypus*. The level of homology of the sex chromosomes between *C*. *angustatus* and the other two *Staurotypus* species remains unknown; therefore, identification of the *C*. *angustatus* sex chromosomes and their linkage groups are needed to clarify the ancestral form of sex chromosomes and the initial step of sex chromosome differentiation in Staurotypinae.

*S*. *triporcatus* and *S*. *salvinii* are the second case of reptilian species for which sex chromosomes were found to have the same origin as the avian Z sex chromosome. The first case is the Hokou gecko (*Gekko hokouensis*), in which six chicken Z-linked genes (*ACO1*, *ATP5A1*, *CHD1*, *DMRT1*, *GHR*, and *RPS6*) were all mapped to the Z chromosome in the same order as that of the ostrich Z chromosome [22], [27]. In *G*. *hokouensis*, the W homolog of *DMRT1* was located in the pericentromeric region where multiple rearrangements including a pericentric inversion occurred. Consequently, recombination should have been suppressed between the Z and W chromosomes. This suggests that functional divergence may have occurred in the W homolog. *DMRT1* is a strong candidate of the sex-determining gene in birds, which is deleted in the chicken W chromosome and also in the W chromosomes of paleognathous birds, emu (*Dromaius novaehollandiae*), double-wattled cassowary (*Casuarius casuarius*), and ostrich [18], [38], and is considered to be involved in testis determination by twofold gene dosage in ZZ males [39], [40]. By contrast, in the African clawed frog (*Xenopus laevis*), a paralog of *DMRT1* located only on the W chromosome, *DM-W*, was identified as the ovary-determinant gene [41]. In *S*. *triporcatus* and *S*. *salvinii*, the X and Y homologs of *DMRT1* were mapped near the secondary constrictions where the X and Y chromosomes might be structurally differentiated. However, the male-specific region on the Y chromosome, which is involved in male sex determination, is still unknown because no intra-chromosomal rearrangement, partial deletion of the Y chromosome, and/or structurally differentiated Y-linked gene has yet been found. Hence, another molecular cytogenetic approach is needed to identify the critical sex-determining region in these species.

## Supporting Information

Figure S1 **Chromosomal locations of ostrich homologs of nine chicken Z-linked genes in female ostrich.** (A, B) FISH pattern of *NARS* on PI-stained metaphase spread (A) and Hoechst-stained pattern of the same metaphase spread (B). (C–N) FISH signals of *ACO1* (C), *RNF20* (D), *DMRT1* (E), *NFIB* (F), *FER* (G), *HMGCR* (I), *KIF2A* (K), and *VCP* (M) on PI-stained Z chromosomes, and FISH signals of *FER* (H), *HMGCR* (J), *KIF2A* (L), and *VCP* (N) on PI-stained W chromosomes. No signals of *ACO1*, *RNF20*, *DMRT1*, and *NFIB* were detected on the W chromosomes. Arrows indicate the hybridization signals of the genes. Scale bars represent 10 µm (A, B) and 2.5 µm (C–N).
(PDF)

Figure S2 **Comparative cytogenetic maps of 16 functional genes on the Z chromosome (SCAZ) and W chromosome (SCAW) of the ostrich (** ***S. camelus*** **, SCA).** The chromosomal locations of seven genes (*TMOD*, *RPS6*, *NTRK2*, *SPIN*, *CHD1*, *GHR*, and *ATP5A1*) written in red were taken from our previous report [27].
(PDF)

Figure S3 **Chromosomal locations of** ***S. salvinii*** **homologs of 16 chicken Z-linked genes in male** ***S. salvinii*** **.** (A, B) FISH pattern of *VCP* on PI-stained metaphase spread (A) and Hoechst-stained pattern of the same metaphase spread (B). (C–Z, A’–E’) FISH signals of *TMOD* (C, D), *ACO1* (E, F), *RNF20* (G, H), *DMRT1* (I, J), *NFIB* (K, L), *RPS6* (M, N), *NTRK2* (O, P), *SPIN* (Q, R), *FER* (S, T), *CHD1* (U), *HMGCR* (V, W), *KIF2A* (X, Y), *GHR* (Z, A’), *ATP5A1* (B’, C’), and *NARS* (D’, E’) on PI-stained X and Y chromosomes. Arrows indicate the hybridization signals of the genes. Scale bars represent 10 µm (A, B) and 2.5 µm (C–Z, A’–E’).
(PDF)

Figure S4 **Comparative cytogenetic maps of 16 functional genes on the X and Y chromosomes of** ***S. triporcatus*** **(STRX and STRY) and** ***S. salvinii*** **(SSAX and SSAY).**
(PDF)

Table S1 **Degenerate oligonucleotide primers used for molecular cloning of** ***S. triporcatus*** **homologs of 16 chicken Z-linked genes.**
(XLS)
