# Supplementary figures and images for: The Staurotypus Turtles and Aves Share the Same Origin of Sex Chromosomes but Evolved Different Types of Heterogametic Sex Determination
Source: PLoS One. 2014 Aug 14;9(8):e105315. doi: 10.1371/journal.pone.0105315 (PMC4133349; doi:10.1371/journal.pone.0105315)

**A****NARS**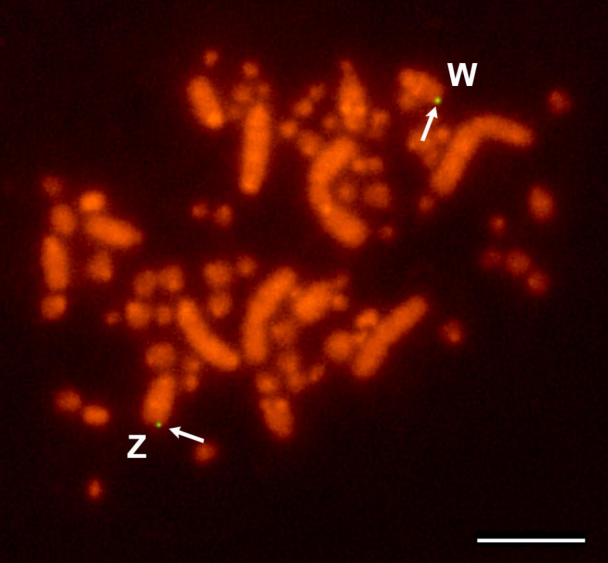**B**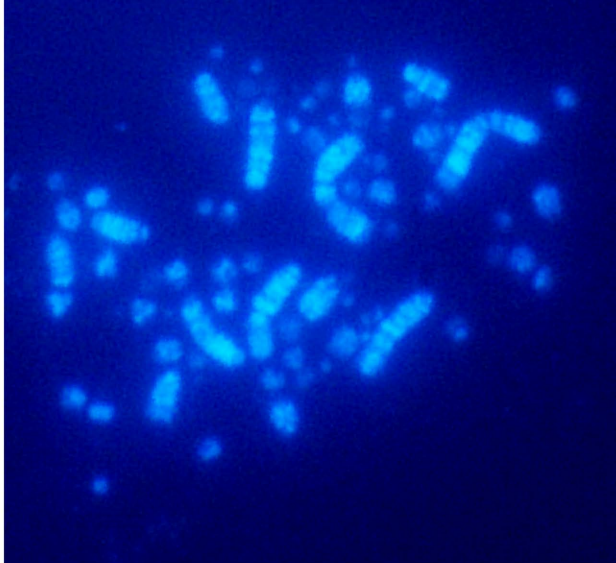**ACO1**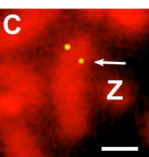**RNF20**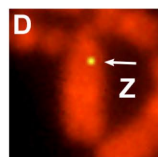**DMRT1**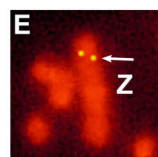**NFIB**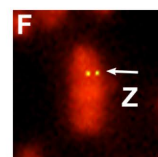**FER**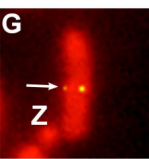**HMGCR**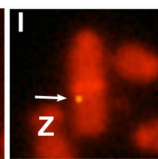**KIF2A**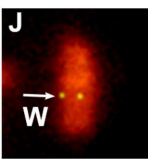**KIF2A**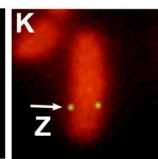**VCP**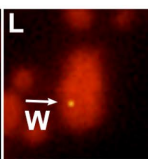**VCP**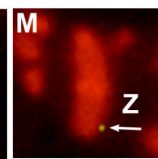**VCP**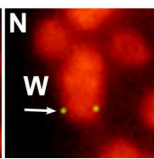

Supplement: Figure S1 — Chromosomal locations of ostrich homologs of nine chicken Z-linked genes in female ostrich. (A, B) FISH pattern of NARS on PI-stained metaphase spread (A) and Hoechst-stained pattern of the same metaphase spread (B). (C–N) FISH signals of ACO1 (C), RNF20 (D), DMRT1 (E), NFIB (F), FER (G), HMGCR (I), KIF2A (K), and VCP (M) on PI-stained Z chromosomes, and FISH signals of FER (H), HMGCR (J), KIF2A (L), and VCP (N) on PI-stained W chromosomes. No signals of ACO1, RNF20, DMRT1, and NFIB were detected on the W chromosomes. Arrows indicate the hybridization signals of the genes. Scale bars represent 10 µm (A, B) and 2.5 µm (C–N). (PDF) [file pone.0105315.s001.pdf]

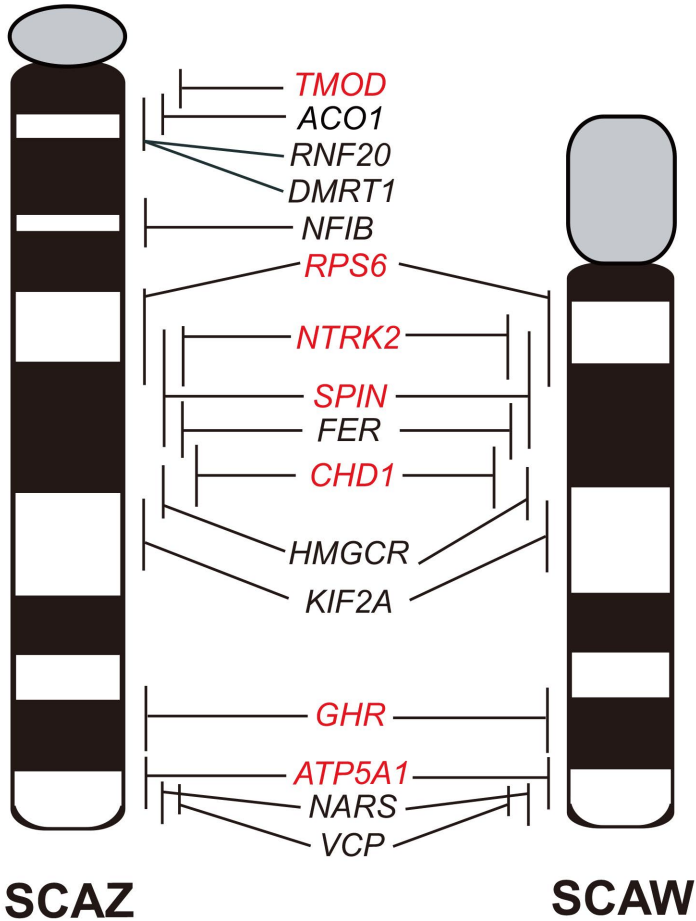

Supplement: Figure S2 — Comparative cytogenetic maps of 16 functional genes on the Z chromosome (SCAZ) and W chromosome (SCAW) of the ostrich ( S. camelus , SCA). The chromosomal locations of seven genes (TMOD, RPS6, NTRK2, SPIN, CHD1, GHR, and ATP5A1) written in red were taken from our previous report [27]. (PDF) [file pone.0105315.s002.pdf]

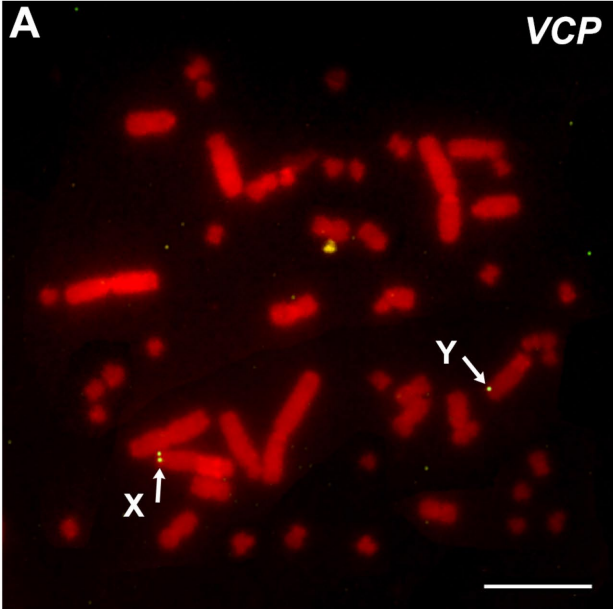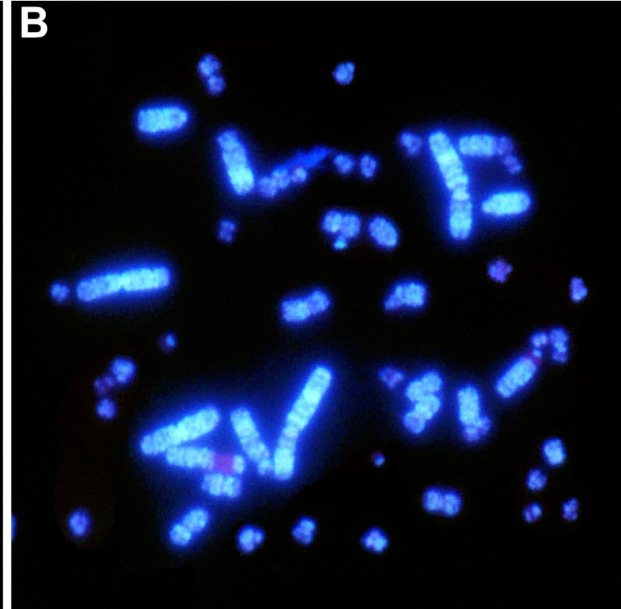

*TMOD* ————— *ACO1* ————— *RNF20* ————— *DMRT1*

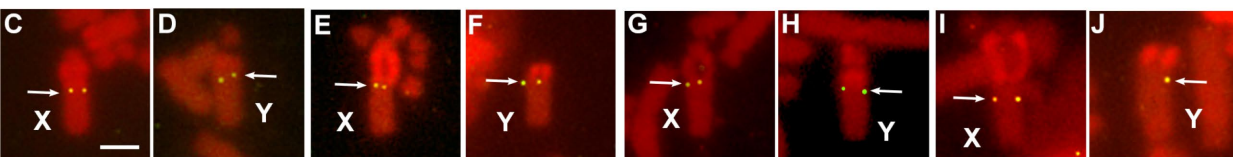

*NFIB* ————— *RPS6* ————— *NTRK2* ————— *SPIN*

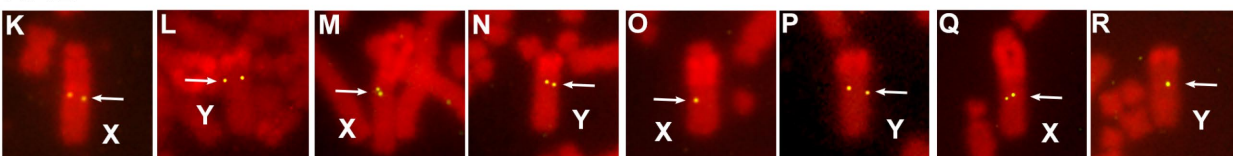

*FER* ————— *CHD1* ————— *HMGCR* ————— *KIF2A*

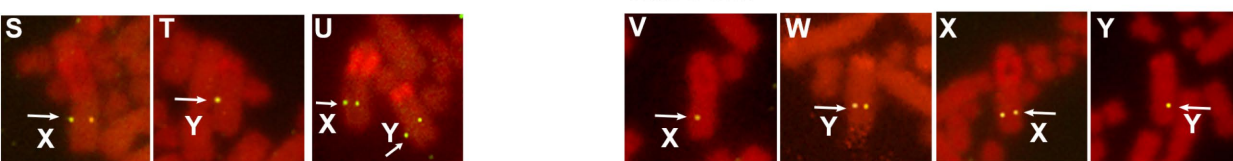

*GHR* ————— *ATP5A1* ————— *NARS*

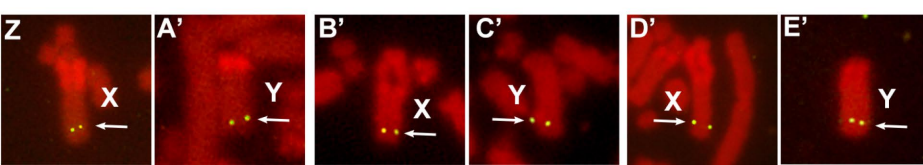

Supplement: Figure S3 — Chromosomal locations of S. salvinii homologs of 16 chicken Z-linked genes in male S. salvinii . (A, B) FISH pattern of VCP on PI-stained metaphase spread (A) and Hoechst-stained pattern of the same metaphase spread (B). (C–Z, A’–E’) FISH signals of TMOD (C, D), ACO1 (E, F), RNF20 (G, H), DMRT1 (I, J), NFIB (K, L), RPS6 (M, N), NTRK2 (O, P), SPIN (Q, R), FER (S, T), CHD1 (U), HMGCR (V, W), KIF2A (X, Y), GHR (Z, A’), ATP5A1 (B’, C’), and NARS (D’, E’) on PI-stained X and Y chromosomes. Arrows indicate the hybridization signals of the genes. Scale bars represent 10 µm (A, B) and 2.5 µm (C–Z, A’–E’). (PDF) [file pone.0105315.s003.pdf]

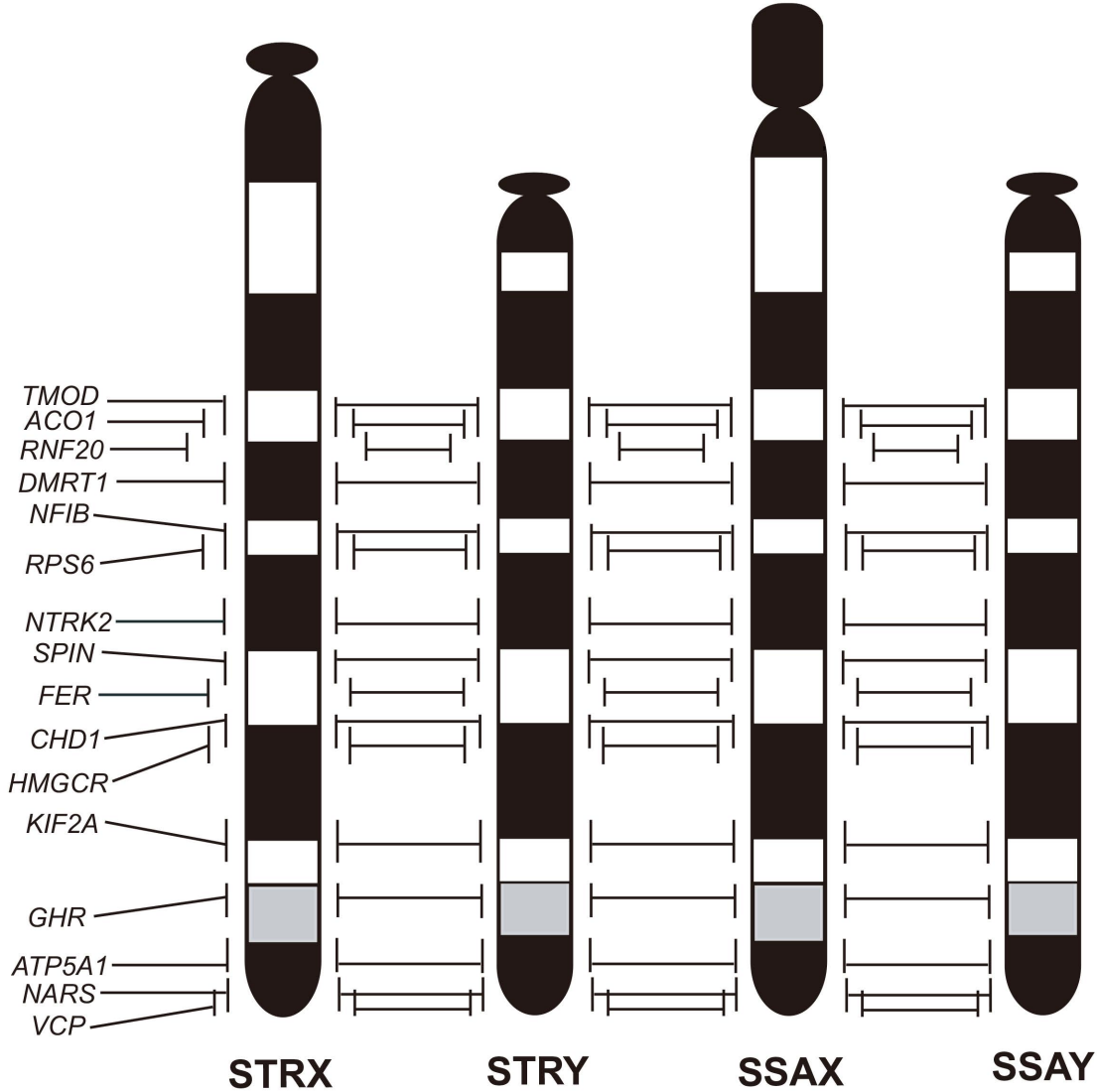

Supplement: Figure S4 — Comparative cytogenetic maps of 16 functional genes on the X and Y chromosomes of S. triporcatus (STRX and STRY) and S. salvinii (SSAX and SSAY). (PDF) [file pone.0105315.s004.pdf]
